# Aerobic Exercise Training Decreases Hepatic Asprosin in Diabetic Rats

**DOI:** 10.3390/jcm8050666

**Published:** 2019-05-12

**Authors:** Jeong Rim Ko, Dae Yun Seo, Tae Nyun Kim, Se Hwan Park, Hyo-Bum Kwak, Kyung Soo Ko, Byoung Doo Rhee, Jin Han

**Affiliations:** 1 National Research Laboratory for Mitochondrial Signaling, Department of Physiology, BK21 Plus Project Team, College of Medicine, Cardiovascular and Metabolic Disease Center, Inje University, Busan 47392, Korea; kjrsos0217@gmail.com (J.R.K.); sdy925@gmail.com (D.Y.S.); kimtn031@gmail.com (T.N.K.); kskomd@paik.ac.kr (K.S.K.); bdrhee@hanmail.net (B.D.R.); 2 Institute of Sports Medicine, Hannam University, Daejeon 34430, Korea; psh8179@gmail.com; 3 Department of Kinesiology, Inha University, Incheon 22212, Korea; kwakhb@inha.ac.kr

**Keywords:** asprosin, PKA, AMPK, TGF-β, aerobic exercise, liver, type 1 diabetes

## Abstract

Asprosin, a novel hormone released from white adipose tissue, regulates hepatic glucose metabolism and is pathologically elevated in the presence of insulin resistance. It is unknown whether aerobic exercise training affects asprosin levels in type 1 diabetes mellitus (T1DM). The aim of this study was to determine whether (1) aerobic exercise training could decrease asprosin levels in the liver of streptozotocin (STZ)-induced diabetic rats and (2) the reduction in asprosin levels could induce asprosin-dependent downstream pathways. Five-week-old male Sprague–Dawley rats were randomly divided into control, STZ-induced diabetes (STZ), and STZ with aerobic exercise training groups (*n* = 6/group). T1DM was induced by a single dose of STZ (65 mg/kg intraperitoneally (i.p.)). The exercise group was made to run on a treadmill for 60 min at a speed of 20 m/min, 4 days per week for 8 weeks. Aerobic exercise training reduced the protein levels of asprosin, PKA, and TGF-β but increased those of AMPK, Akt, PGC-1β, and MnSOD. These results suggest that aerobic exercise training affects hepatic asprosin-dependent PKA/TGF-β and AMPK downstream pathways in T1DM.

## 1. Introduction

Diabetes mellitus, a global epidemic disease, is the most common metabolic disorder caused by the impairment of insulin secretion and glucose metabolism [1]. Type 1 diabetes mellitus (T1DM) is characterized by a deficiency in insulin secretion from β-cells, resulting in hyperglycemia and impaired glucose metabolism [2]. Abnormal glucose metabolism promotes liver injury by disturbing the balance between glucose production and uptake, indicating upregulation of G protein-dependent cyclic adenosine monophosphate (cAMP) and protein kinase A (PKA) in the liver [3,4]. However, the role of the cAMP/PKA signaling pathway in the liver of streptozotocin (STZ)-induced diabetic models remains unclear.

Asprosin has been identified as a novel hormone which is cleaved from the C-terminal end of profibrillin, released from white adipose tissue [5]. Recent studies in mice and humans demonstrated that asprosin is closely related to glucose and insulin production in the liver during fasting and its presence is a pathological risk factor for insulin resistance [5,6,7]. Circulating asprosin also results in metabolic syndrome, while a reduction in asprosin levels protects against hyperinsulinemia related to metabolic syndrome [5]. Greenhill et al. [7] found that asprosin administration to mice increased blood glucose levels. In contrast, inhibition of asprosin or genetic deletion of profibrillin (*FBN 1*) decreased the plasma levels of insulin and the production of glucose in the liver in vivo, indicating that asprosin plays a role in fasting-induced hepatic glucose production [8]. Furthermore, increased asprosin levels in the liver promote cAMP and PKA signaling pathways, which results in acceleration of glucose release in the circulation. The activation of the cAMP/PKA signaling pathways negatively regulates glucose production through AMP-activated protein kinase (AMPK) inactivation in humans and animals [9,10]. The existing body of research on AMPK, an exercise-induced major energy sensor, suggests that it contributes to the improvement of impaired glucose metabolism in diabetes [11]. Our previous study has shown that aerobic exercise training activates AMPK by increasing peroxide proliferator-activated receptor gamma coactivator 1-alpha (PGC-1α), fibronectin type III domain-containing protein 5 (FNDC5), and uncoupling proteins (UCPs), suggesting that these AMPK signaling pathways improve insulin resistance and glucose intolerance in STZ-induced T1DM rats [12]. However, the signaling pathway downstream of asprosin in the liver of T1DM rats during aerobic exercise training remains to be elucidated.

Exercise is a cornerstone in the management of diabetes as it changes the dynamics of glucose and affects its metabolism in the liver [13]. Aerobic exercise training improves hepatic glucose metabolism via inhibition of cAMP/PKA and activation of AMPK signaling pathways, suggesting that aerobic exercise training may decrease asprosin levels in diabetes [14]. In contrast, Wiecek et al. [15] found that a single anaerobic exercise session increased plasma asprosin levels but did not find a correlation between asprosin and glucose levels in women. A systematic understanding of how asprosin contributes to glucose production in the liver during aerobic exercise training is still lacking.

Herein, we investigated the effect of aerobic exercise training on hepatic asprosin levels in STZ-induced diabetic rats. Furthermore, we examined the effects of asprosin on PKA/AMPK/TGF-β-mediated pathway. This work provides new insights into novel mechanisms of how aerobic exercise-modulated hepatic asprosin level controls asprosin-dependent downstream pathways for diabetes treatment.

## 2. Materials and Methods

### 2.1. Animal Care

This study was approved by the Institutional Animal Use and Care Committee of Inje University (Busan, Korea) [13]. Five-week-old male Sprague–Dawley (SD) rats (*n* = 18) were purchased from Orient Bio (Gyeonggi-do, Korea). Two rats were placed in a single cage until they were used for the experiments. The number of biological replicates in each group was six, which was the minimal number to determine statistical significance. Food and water were provided ad libitum. Humidity and temperature in the animal room were maintained at 40–60% and 23 °C, respectively, with a 12/12 h dark/light cycle [13].

### 2.2. Diabetes Model

The 18 SD rats were divided into control (CON), STZ-induced diabetes (STZ), and STZ with exercise training (STZ + EXE) groups. The diabetes model rats (*n* = 12) were induced by an intraperitoneal injection of 65 mg/kg STZ (Sigma-Aldrich, Oakville, ON, Canada). The same amount of sodium citrate buffer solution (5.5 mL/kg, Sigma-Aldrich) was injected to the control group (*n* = 6). Blood glucose level was measured at 72 h after injection (Built Technology Co., Ltd., Nanjing, China). Then diabetic rats with a fasting plasma glucose level > 250 mg/mL were selected for subsequent experiments [13].

### 2.3. Aerobic Exercise Training

**Figure 1:**
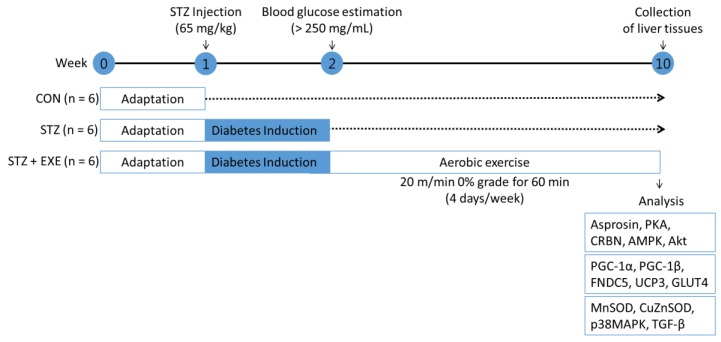
Schematic diagram of the experimental timeline. CON: control, STZ: streptozotocin, EXE, exercise.

After confirming the fasting glucose levels, the rats were mad to run on a treadmill at 20 m/min for 60 min per day, 4 days per week, for 8 weeks (Eco 3/6 treadmill; Columbus Instruments, Columbus, OH, USA) [16] (Figure 1). On the day after the final aerobic exercise training, all sampling procedures were performed under anesthesia using intraperitoneal injections of alfaxan (25 mg/kg). The liver tissues were refrigerated in liquid nitrogen and stored at −80 °C until analysis.

### 2.4. Western Blotting

Proteins were extracted with lysis buffer (50 mM HEPES, 100 mM NaF, 10 mM EDTA, 50 mM Na pyrophosphate, 1% Triton at pH 7.4, and 10 mM Na Orthovanadate) for 15 min at 4 °C as previously reported [17]. The total protein samples (25 µg) were subjected to 9% SDS PAGE, transferred onto an activated polyvinylidene difluoride membrane (Immobilon P, Millipore, Denmark), and incubated overnight at 4 °C with primary antibodies followed by peroxidase-conjugated secondary antibodies. The signal was detected by Super Signal West Pico Chemiluminescent Substrate (Thermo Fisher Scientific, San Jose, CA, USA). Images were obtained using a densitometer (Bio-Rad Laboratories, Hercules, CA, USA) and resolved quantitatively. Each band level was standardized with respect to the β-actin control. Anti-asprosin (AdipoGen, Seoul, Korea), anti-PKA, anti-GLUT4, anti-SOD1, anti-SOD2, anti-β-actin antibodies (Santa Cruz, CA, USA), anti-CRBN (Sigma-Aldrich, Louis, MO, USA), anti-AMPK, anti-Akt, anti-p38MAPK, anti-TGF-β (Cell Signaling Technology, Beverly, MA, USA), anti-FNDC5, anti-PGC-1α, and anti-UCP3 antibody (Abcam, Cambridge, MA, USA) were used.

### 2.5. Statistical Analysis

All results are shown as mean ± standard error (SE). Statistical differences were assessed by ANOVA and Tukey’s multiple comparisons using the SPSS 25.0 program (SPSS, Inc., Chicago, IL, USA). Values of *p* < 0.05 were considered statistically significant.

## 3. Results

### 3.1. Aerobic Exercise Training Decreased Asprosin and PKA Levels but Increased AMPK and Akt Levels in the Liver of T1DM Rats

**Figure 2:**
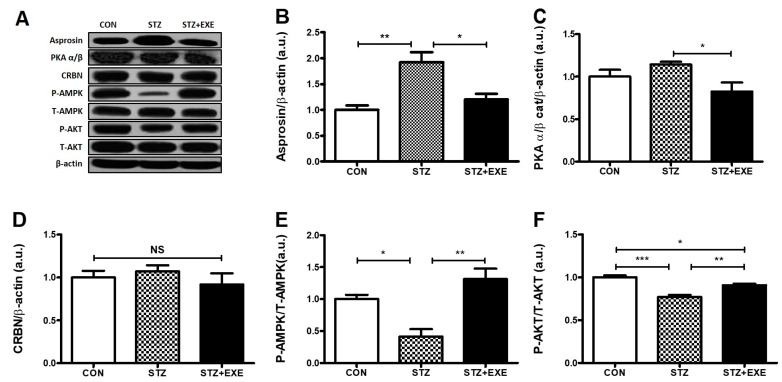
Aerobic exercise training decreased asprosin and PKA levels, but increased AMPK and Akt levels in the liver of type 1 diabetic rats. (**A**) Representative western blot, (**B**) Western blot analysis of liver asprosin levels in type 1 diabetic rats, (**C**) Western blot analysis of PKA levels in type 1 diabetic rats, (**D**) Western blot analysis of CRBN levels in type 1 diabetic rats, (**E**) Western blot analysis of AMPK levels in type 1 diabetic rats, (**F**) Western blot analysis of Akt levels in type 1 diabetic rats. Results are expressed as the mean ± standard error (SE); * *p* < 0.5, ** *p* < 0.05, and *** *p* < 0.001; NS, not significant.

To determine whether aerobic exercise training influences hepatic asprosin and PKA levels, the rats were made to run on a treadmill 60 min/day, 4 days/week, for 8 weeks. Interestingly, we confirmed that the level of asprosin in the STZ group was significantly higher than in the CON group (*p* < 0.005), while the level of asprosin in the STZ + EXE group was significantly lower than in the STZ group (*p* < 0.05, Figure 2B). Furthermore, the level of PKA, the downstream target of asprosin [5], in the STZ + EXE group was significantly lower than in the STZ group (*p* < 0.05, Figure 2C). No significant difference in CRBN among the three groups was observed (Figure 2D). The level of AMPK, which was blocked by PKA [18] and directly affected by CRBN [13], in the STZ group was significantly lower than in the CON group (*p* < 0.05), while the level of AMPK in the STZ + EXE group was significantly higher than in the STZ group (*p* < 0.005, Figure 2E). The level of Akt, which interacts with AMPK [19], significantly increased in the STZ + EXE groups as compared to the STZ group (*p* < 0.05, Figure 2F). These results suggest that aerobic exercise training ameliorated hepatic asprosin-dependent downstream pathways in T1DM.

### 3.2. Aerobic Exercise Training Increased PGC-1β Levels in the Liver of T1DM Rats

**Figure 3:**
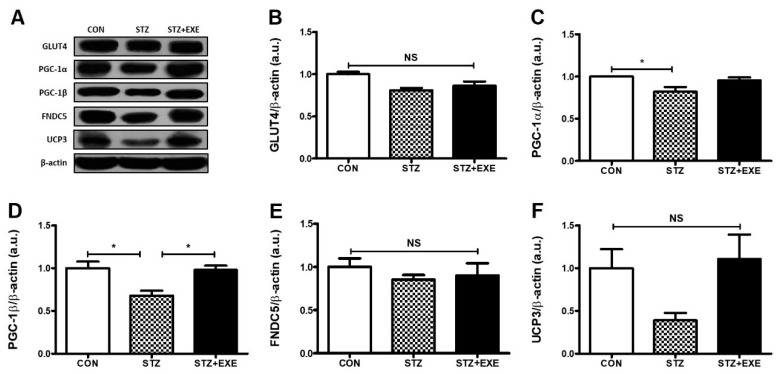
Aerobic exercise training increased PGC-1β levels in the liver of type 1 diabetic rats. (**A**) Representative western blot. (**B**) Western blot analysis of liver GLUT-4 levels in type 1 diabetic rats, (**C**) Western blot analysis of liver PGC-1α levels in type 1 diabetic rats (**D**) Western blot analysis of liver PGC-1β levels in type 1 diabetic rats (**E**) Western blot analysis of liver FNDC5 levels in type 1 diabetic rats, (**F**) Western blot analysis of liver UCP3 levels in type 1 diabetic rats. Results are expressed the mean ± SE. * *p* < 0.5; NS, not significant.

To determine whether decreased hepatic asprosin and increased AMPK levels modulate glucose-regulated signaling pathways, we examined the levels of GLUT4, PGC-1α, PGC-1β, FNDC5, and UCP3 in the liver of T1DM rats following 8 weeks of aerobic exercise training. We found that the level of GLUT4 showed no significant change in any group (Figure 3B). The level of PGC-1α in the STZ group was significantly lower than in the CON group (*p* < 0.05), but there was no significant difference in the level of PGC-1α between the STZ and the STZ + EXE group (Figure 3C). The level of PGC-1β in the STZ + EXE group was significantly higher than in the STZ group (*p* < 0.05, Figure 3D). The levels of FNDC5 and UCP3 showed no significant difference in any group (Figure 3E,F). These results suggest that hepatic asprosin and AMPK levels regulate PGC-1β levels in the liver of T1DM rats.

### 3.3. Aerobic Exercise Training Increased MnSOD Levels and Decreased TGF-β Levels in the Liver of T1DM Rats

**Figure 4:**
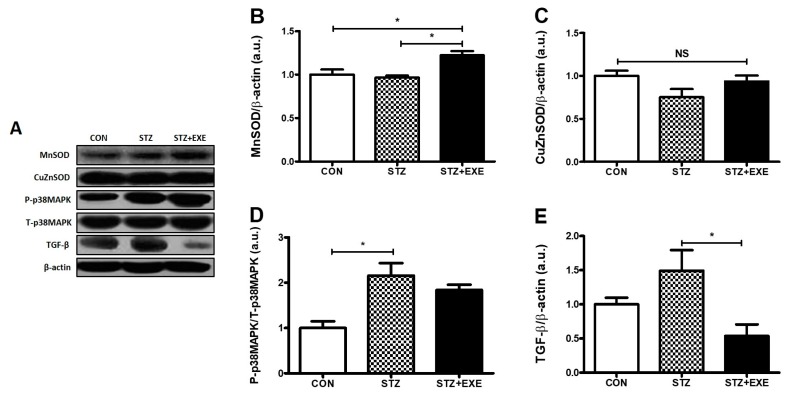
Aerobic exercise increased MnSOD levels and decreased TGF-β levels in the liver of type 1 diabetic rats. (**A**) Representative western blot. (**B**) Western blot analysis of liver MnSOD, (**C**) Western blot analysis of liver CuZnSOD, (**D**) Western blot analysis of liver p-38MAPK, (**E**) Western blot analysis of liver TGF-β. Results are expressed as the mean ± SE. * *p* < 0.5; NS, not significant.

The level of MnSOD, an antioxidant enzyme [20], in the STZ + EXE group was significantly higher than in the STZ group (*p* < 0.05, Figure 4B), but the levels of CuZnSOD showed no significant difference in any group (Figure 4C). The level of p-38MAPK in the STZ + EXE group was significantly higher than in the STZ group (*p* < 0.05, Figure 4D). The level of TGF-β, a fibrogenic growth factor [21], was decreased in the STZ + EXE group as compared to the STZ group (*p* < 0.05, Figure 4E). These results suggest that aerobic exercise training also improves the antioxidant and anti-fibrosis functions in the liver of T1DM rats.

## 4. Discussion

The present study examined whether aerobic exercise training could decrease asprosin levels in the liver of STZ-induced diabetic rats and whether exercise-modulated asprosin could contribute to asprosin-dependent pathways leading to reduced hepatic glucose release. The major findings were that aerobic exercise training decreased asprosin/PKA/TGF-β levels but increased AMPK, Akt, PGC-1β, and MnSOD levels in the liver of STZ-induced diabetic rats. These results reveal that aerobic exercise training ameliorates hepatic asprosin-dependent downstream pathways in T1DM. This report suggests that aerobic exercise-modulated hepatic asprosin could be a potential target for the treatment of T1DM.

T1DM is characterized by a lack of insulin secretion from pancreatic β-cells. This condition is known to play a role in hepatic glucose metabolism [22]. However, the underlying mechanism of hepatic glucose metabolism dysfunction under constant high levels of blood glucose has not been completely investigated. Interestingly, recent reports have demonstrated that asprosin is a novel hormone present at high levels in obesity and fasting conditions. In addition, asprosin levels have been found to be positively correlated with hepatic gluconeogenesis and high blood glucose levels, resulting in increased asprosin levels in type 2 diabetes [5]. Furthermore, Wang et al. [23] found that an elevation in asprosin level causes the increase of blood glucose, insulin resistance, and waist circumference. These results suggest that asprosin could be a biomarker for the early diagnosis of diabetes. On the basis of these observations, we hypothesized that the liver of STZ-induced diabetic rats contributes to high blood glucose level owing to the elevation of asprosin level. We found that STZ-induced diabetic rats have increased blood glucose levels [13] and hepatic asprosin levels (Figure 2B). These findings suggest that the increased hepatic asprosin level leads to impairment of hepatic glucose metabolism in diabetes. In line with previous reports, Romere et al. [5] observed that recombinant asprosin treatment significantly increased blood glucose levels. On the basis of these results, we next confirmed the effect of aerobic exercise training on blood glucose levels and hepatic asprosin level. These results suggest that aerobic exercise training suppresses blood glucose level through the reduction of hepatic asprosin levels in STZ-induced diabetic rats. Another study on blood suggested that a single anaerobic exercise session contributes to increased asprosin levels in women [16]. This discrepancy may be attributed to differences in exercise type, participants, and evaluated parameters.

Hepatic asprosin has been shown to increase the activity of PKA, a secondary mediator of cAMP, resulting in an impairment of glucose metabolism in the liver [5,10]. Previous studies showed that a PKA-mediated pathway contributes to the suppression of AMPK activation, involved in the regulation of glucose metabolism [24,25,26]. Indeed, activation of AMPK induces the upregulation of Akt and PGC-1β, suggesting that these signaling pathways play a key role in the prevention of diabetes [27]. Pan et al. also [28] found that PKA augments TGF-β activation. TGF-β/SMAD3 signaling pathway is important in regulating glucose and energy homeostasis and might play a role in the chronic complications of diabetes [29]. TGF-β serves to activate hepatic gluconeogenesis by counteracting LKB1–AMPK signaling inactivation through dephosphorylation. AMPK-mediated suppression of gluconeogenesis involves the exit of FoxO1 from the nucleus to the cytoplasm via AMPK-mediated phosphorylation on multiple sites [30]. Collectively, these studies suggest that PKA-mediated pathway negatively regulates AMPK, Akt, and PGC-1β activation and positively regulates TGF-β activation for glucose metabolism in the liver of T1DM rats [31,32]. In the present study, we determined whether aerobic exercise training is suitable for the downregulation of PKA and TGF-β and the upregulation of AMPK, Akt, and PGC-1β in the liver of STZ-induced diabetic rats. As expected, we found that aerobic exercise training decreases PKA and TGF-β and increases AMPK, Akt, and PGC-1β (Figure 2E,F, Figure 3D, and Figure 4E) levels. It is possible that a direct interaction between aerobic exercise-modulated asprosin-dependent PKA/TGF-β and AMPK downstream pathways occurs in the liver of STZ-induced rats.

We acknowledge that these are preliminary findings, and this study has several limitations that should be considered when conducting related studies in the future. First, we did not measure circulating asprosin levels before and after exercise intervention. These might have enabled the determination of a causal relationship among exercise, plasma and hepatic asprosin levels, and hepatic glucose release. T1DM, like type 2 diabetes, may be linked to dysfunctions of the adipose tissue, which may lead to inappropriate secretion of adipokines such as asprosin. However, to the best of our knowledge, for the first time, we showed that hepatic asprosin levels may influence the effective regulation of plasma glucose levels in a T1DM rat model. Second, it is necessary to determine the mechanism underlying the beneficial effect of aerobic exercise training on hepatic asprosin-dependent pathways by focusing on the alterations in insulin- and glucagon-related pathways and in cAMP. Finally, further studies are necessary to elucidate the influence of aerobic exercise-modulated asprosin levels in adipose tissue, skeletal muscle, and blood using insulin-deficient animal models, in order to elucidate its role in the treatment of diabetes.

## 5. Conclusions

**Figure 5:**
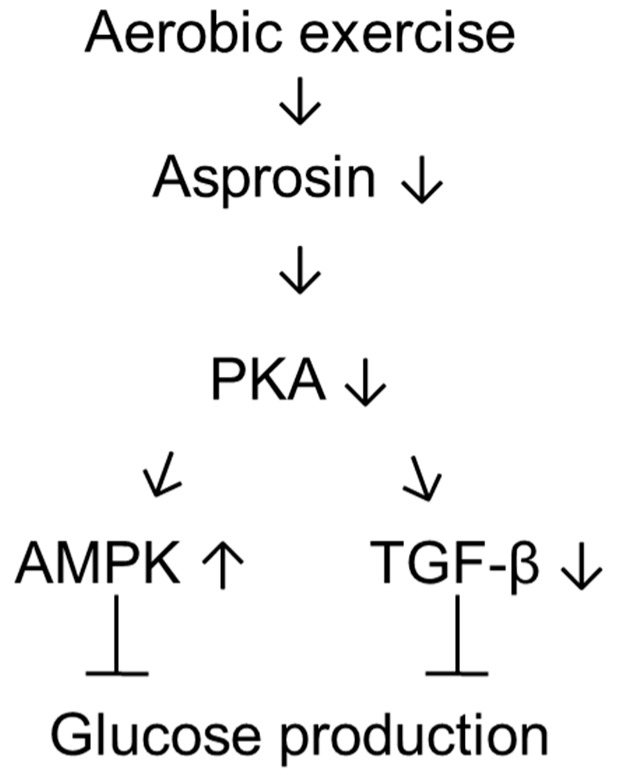
Schematic diagram of the possible mechanisms activated by asprosin in the liver of type 1 diabetic rat following aerobic exercise training.

To the best of our knowledge, this is the first study demonstrating that aerobic exercise training decreased hepatic asprosin level, which led to the amelioration of diabetes-related parameters in T1DM rats. The regulation of pathways downstream of asprosin, such as the regulation of an AMPK/TGF-β-related pathway and a parallel mitochondria-related pathway through PKA, might be beneficial for the treatment of T1DM (Figure 5).
